# Common capacity for far-red light photosynthesis in a canyon thermophilic freshwater system

**DOI:** 10.1007/s00792-026-01422-9

**Published:** 2026-02-21

**Authors:** Ivan J. Moreno, Alexander Bogdanov, Brian Palenik

**Affiliations:** 1 Scripps Institution of Oceanography, University of California, San Diego, La Jolla, CA 92093-0202 USA; 2 Present Address: University of California, Santa Barbara, Santa Barbara, CA 93106 USA

**Keywords:** Cyanobacteria, Photoacclimation, Far-red light, Hot springs, Photosynthesis, FaRLiP

## Abstract

Photosynthetic life is based on absorbing sunlight and turning it into biologically usable energy. In many cases however, canopy-like structures and cavern-like habitats in terrestrial environments can limit the intensity and alter the spectra of light. One acclimation to use filtered light in the near infrared range, typically between 700 and 800 nm is named far-red light photoacclimation or FaRLiP as in recent studies of cyanobacteria. Here we report the common capacity for FaRLiP in the dominant cyanobacterial genera in a canyon hot spring microbial mat ecosystem. We identified FaRLiP in the genomes of cyanobacterial isolates and the metagenomes of mat samples. We show using absorption spectroscopy and HPLC that under far red-light specific isolates show an increase in far red-light absorption and the presence of Chl *f*. Springs in narrow canyons are a microniche where FaRLiP seems highly ecologically advantageous.

**Supplementary Information:**

The online version contains supplementary material available at 10.1007/s00792-026-01422-9.

## Introduction

Cyanobacteria are believed to have evolved the ability to generate oxygen on Earth 2–3 billion years ago, leading to the Great Oxygenation Event and the emergence of diverse cyanobacteria now found ubiquitously across the globe (Olejarz et al. 2021). As environmental conditions changed drastically during this time in Earth’s history, cyanobacteria likely evolved oxidative stress response mechanisms, changes in cell morphology, and ultimately expanded into the many habitats they now occupy ( Sánchez-Baracaldo et al. 2005; Sánchez-Baracaldo and Cardona 2020).

While cyanobacteria are traditionally understood to absorb mainly visible wavelengths of light, recent findings have revealed that the ability to absorb light past the 700 nm range may be prevalent in cyanobacteria from several phylogenetic groups (Gan et al. 2014a; Ho et al. 2016; Antonaru et al. 2020). While the molecular biology of far-red light photosynthesis (FaRLiP) has advanced rapidly, there are fewer studies on where this type of photosynthesis may be ecologically relevant. Specific aquatic and terrestrial environments presumably have the light conditions that help determine the presence of this FaRLiP phenotype. Other recent studies have focused on the astrobiological implications of FaRLiP which include the ability of FaRLiP cyanobacteria to adapt to a wide range of extreme environments where light may be limited such as on exoplanets (Jung et al. 2023).

Microbial mats have long been used as analogs to study early microbial life due to their morphological similarities to well-studied ancient (and modern) stratified and calcified structures called stromatolites such as those seen in Shark Bay, Australia. These have been found to contain far-red light absorbing cyanobacteria (Chen et al. 2012). Investigations into microbial mats in hot springs of Yellowstone National Park, USA, have revealed the presence of cyanobacteria that are both filamentous and single celled, with the former being a FaRLiP cyanobacterium (Gan et al. 2014b). Other biofilms that contain cyanobacteria with the capacity for FaRLiP include endoliths of beachrocks. These have been shown to make up to 20% of the photosynthesis rates in their respective communities (Trampe and Kühl 2016; Kühl et al. 2020). Molecular genetic studies have shown that a cluster of genes, *psbA3, apcA2, apcB2, apcD2, apcE2, apcD3, psbD3, pdbC2, psbB2, psbH2, rfpB, rfpA, rpfC, chlf, psaA2, psaB2, psaL2, psaI2, psaF2* and *psaJ2*, are responsible for this phenotype in the thermophilic freshwater cyanobacterium *Leptolyngbya* sp*.* JSC-1, named after the group of researchers who described it at the NASA Johnson Space Center (Brown et al. 2010; Gan et al. 2014b). Genes responsible for the production and utilization of chlorophyll *f* (Chl *f*) include *psa* genes for core subunits of photosystem I, *psb* genes for core subunits of photosystem II, *apc* genes for core subunits of the phycobilisomes, and *rfp* genes for the response regulators RfpB and RfpC, and the phytochrome protein for the photoreceptor master control element, RfpA, which initiates the acclimation into the FaRLiP phenotype (Gan et al. 2014a).

Black Canyon of the Colorado River, located near Hoover Dam, is host to diverse microbial mat communities based on freshwater hot springs (Moreno et al. 2023). These microbiomes are predominantly cyanobacterial, and we found that some strains are closely related to several FaRLiP cyanobacteria previously described in the literature, such as the filamentous thermophilic freshwater cyanobacterium *Leptolyngbya* sp. JSC-1 mentioned previously (Broun et al. 2010; Brown et al. 2010; Gan et al. 2014b). Cyanobacteria from the group *Fischerella* also contain the genes for FaRLiP (Gan et al. 2014a) and these genera were found in Black Canyon (Moreno et al. 2023). We hypothesized that FaRLiP in cyanobacteria would be advantageous in small, narrow, and light-limited canyons such as those found at Arizona Hot Springs within Black Canyon of the Colorado River.

As part of earlier work, we obtained 20 isolates of cyanobacteria and 21 metagenomes from Arizona Hot Springs, with 5 of those isolated cyanobacteria from an initial study surveying the microbiome of the mats found at the hot springs throughout Black Canyon (Moreno et al. 2023). The remaining 15 isolated cyanobacteria and all metagenome microbial mat samples were recovered during a 2019 sampling expedition briefly described below and will be further discussed in a manuscript in preparation. We examine here what these data tell us about the presence of FaRLiP in this ecosystem. We demonstrate that FaRLiP may be more prevalent than previously thought and support this by demonstrating that some isolated cyanobacteria grown under far-red light produce chlorophyll *f* (Chl *f*), one of the pigments associated with FaRLiP.

## Methods

### Sampling location

**Fig. 1:**
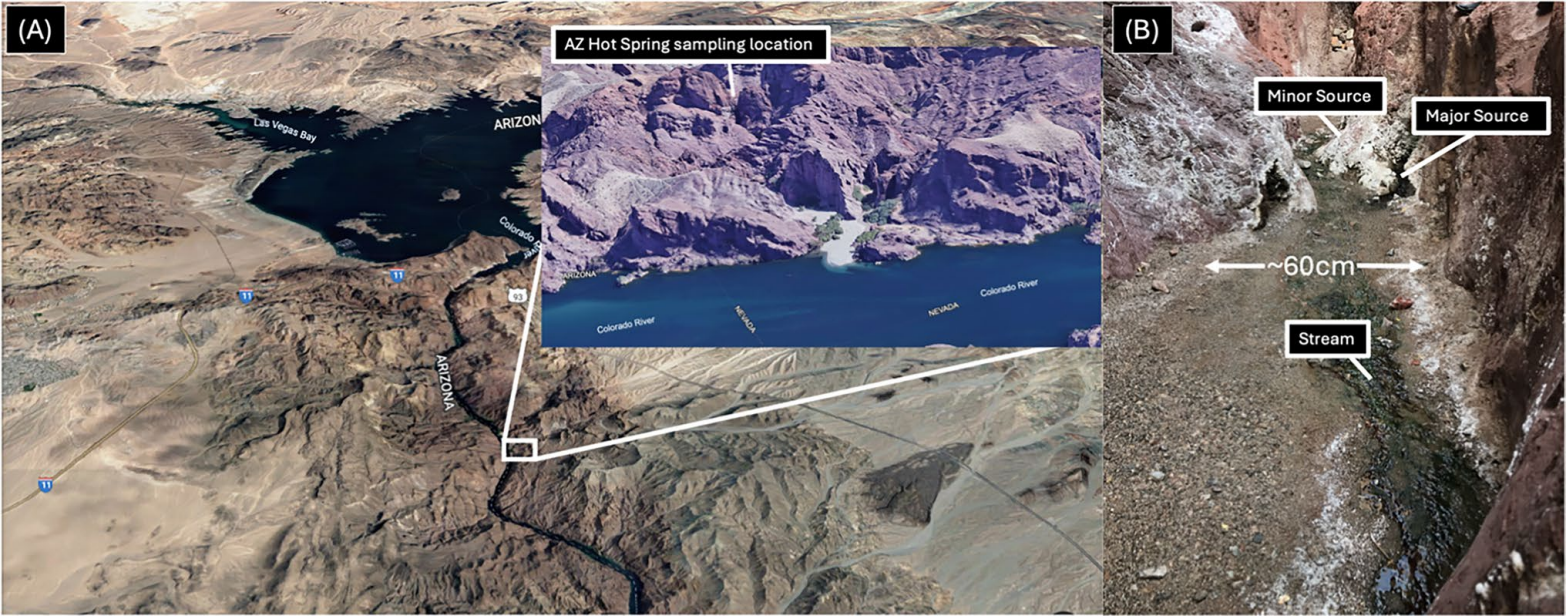
**A** Map displaying the location of Arizona Hot Springs at Black Canyon of the Colorado River, located along the Arizona and Nevada border. **B** Digital picture of the sampling location for this study found along the AZ hot spring trail. Measurements shown to display approximate width of canyon at sampling location

Coordinates of samples recovered from Arizona Hot Springs are 35.96055256257244, -114.7253717552983. An aerial view of the canyon created using Google Earth can be seen in Fig. 1A along with a photocomposite, showing the narrowness of the canyon (60 cm wide) where the samples and most of the isolated cyanobacteria for this study were obtained. Isolates with prefix BC15- and BC16-, representing Black Canyon and the year of collection (2015 and 2016, respectively) were collected and described previously in (Moreno et al. 2023). All microbial mat samples were recovered from the Arizona Hot Springs location in samples as seen in Fig. 1B and replicate mat samples from the same sites were collected for DNA extraction and sequencing. These were used for the construction of metagenome assembled genomes. All mats are described by color with the exception being “minor source”, which was collected from a separate, smaller hot spring that contained a mat sample that had patches of colors that were green and brown at the surface level.

### Genomics

**Fig. 2:**
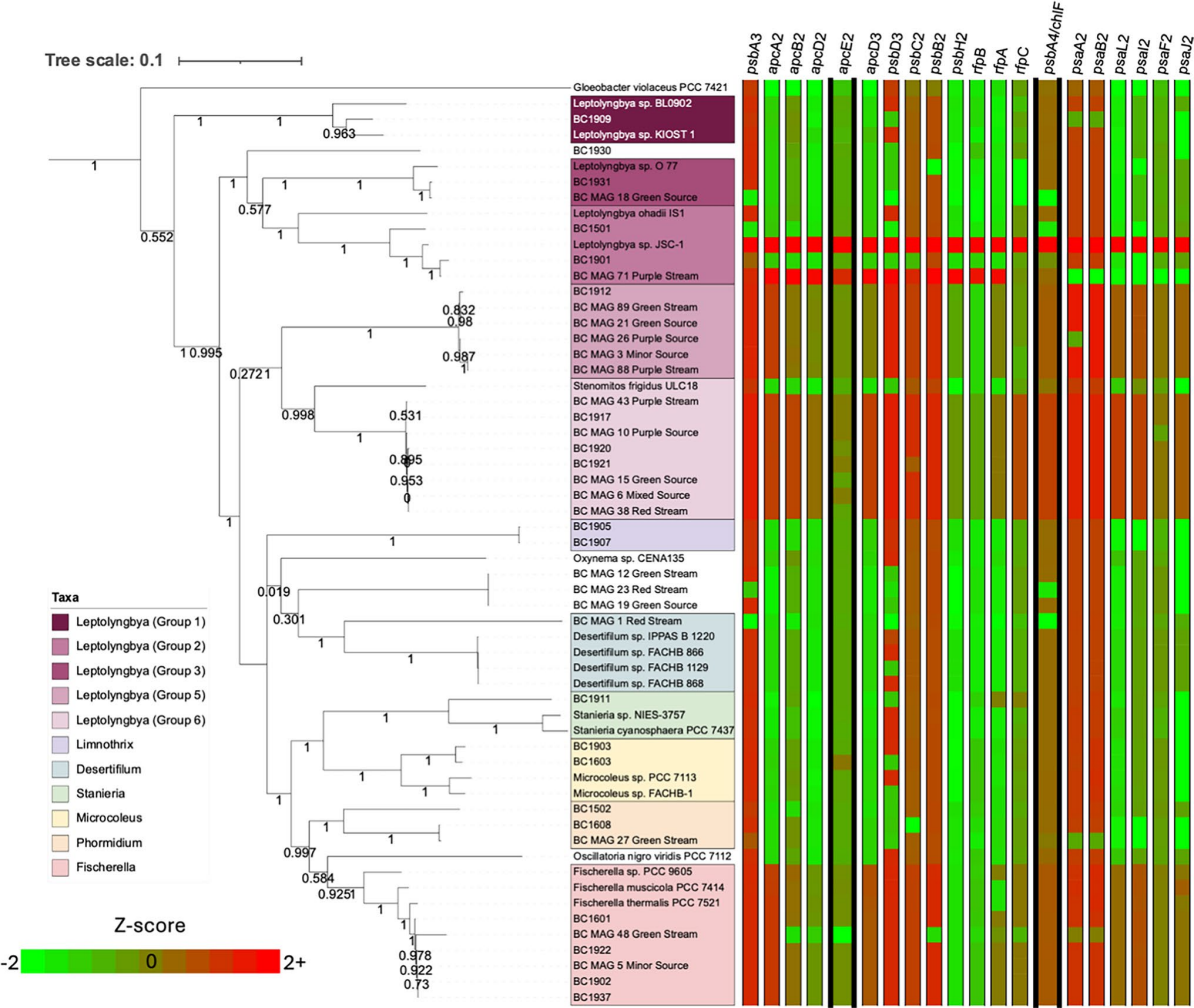
Clusters of Orthologous Groups. Z-scores for genes identified as part of the FaRLiP cluster of *Leptolyngbya* JSC-1. Negative infinity is included as the lowest possible z-score as those genomes missing genomes were given the lowest possible z-scores due to using zero as the bit score when calculating z-score. Metagenome assembled genome named BC MAG 14 from Minor Source excluded from tree due to low genome completion value based on CheckM. Marker genes used for FaRLiP prediction in previous studies (*apcE2* and *psbA4*/*chl f*) are indicated by bold columns

Genomes of the cyanobacteria from Black Canyon were previously assembled from cultured isolates of each strain (manuscript in preparation). In a KBase.com narrative, a BLASTp search using the genes in the FaRLiP operon found in strain *Leptolyngbya* JSC-1 were used as a reference to determine the relatedness of each Black Canyon strain to the known Chl *f* producer JSC-1 (Gan et al. 2014a). Genes *psbA3, apcA2, apcB2, apcD2, apcE2, apcD3, psbD3, psbC2, psbB2, psbH2, rfpB, rfpA, rpfC, chlf, psaA2, psaB2, psaL2, psaI2, psaF2* and *psaJ2* were used to check for amino acid bit score using the BLASTp program version 2.13.0 and only the highest scores for each gene were kept. When the search did not return a result, the values were left blank. Each bit score was then converted to a z-score. Those without a bit score were assigned the lowest possible z-score by dividing the average by the standard deviation of the bit scores, with the lowest possible z-score being negative infinity or indicating that there was no gene found (Fig. 2.). Statistics for the genomes assembled from isolated Black Canyon cyanobacteria and those assembled from environmental samples as metagenome assembled genomes (MAGs) can be seen in Tables 1 and 2, respectively.NCBI BioProject ID PRJNA1419906 contains all genomes and metagenomes discussed in this paper.

**Table 1:** Details characterizing the Black Canyon isolated strain genomes used in this study

Isolate ID	Recovered From	Genus Level GTDB Classification	Genome Size (bp)	GC%	CheckM Completeness %	CheckM Contamination %	Chl-F predicted?	FaRLiP cluster?	Live cell absorption @ > 700 nm?	HPLC ChlF
BC1909	AZ hot springs wall minerals	g__Nodosilinea	5,528,265	57.49	97.28	1.81	N	N	–	–
BC1930	Upper source green	g__JACYMB01	5,647,028	51.93	100	0.59	N	N	–	–
BC1931	Upper source green	g__O-77	5,202,101	56.54	98.58	0.94	N	N	–	–
BC1501	*Lower AZ hot springs	g__Elainella	7,259,317	49.24	99.46	1.18	N	N	–	–
BC1901	Upper source purple	g__Elainella	6,205,131	51.10	97.83	0.63	Y	N	+	+
BC1912	Weeping wall cave	g__DSRU01	5,597,316	50.47	99.37	0.27	Y	Y	+	+
BC1917	Upper source purple	g__Leptothermofonsia	5,538,298	50.36	99.53	0.47	Y	Y	+	+
BC1920	AZ main pool	g__Leptothermofonsia	5,653,332	50.32	99.37	0.54	Y	Y	–	–
BC1921	Weeping wall wave	g__Leptothermofonsia	5,496,424	50.40	97.64	0.47	Y	Y	+	+
BC1905	AZ hot springs ladder	g__CACIAM-69d	4,791,316	56.52	98.28	0.27	N	N	–	–
BC1907	AZ hot springs ladder	g__CACIAM-69d	4,734,960	56.53	98.28	0.27	N	N	untested	untested
BC1911	AZ hot springs wall minerals	g__LEGE-06147	6,006,516	41.36	99.56	1.16	N	N	–	–
BC1903	Red stream	g__Allocoleopsis	9,841,780	46.06	99.04	1.74	N	N	–	–
BC1603	*Fluorescent Green Pool 3	g__Allocoleopsis	8,678,288	46.15	99.78	3.3	N	N	–	–
BC1502	*Lower AZ hot springs	g__FACHB-1375	8,679,150	44.22	99.37	0.74	N	N	–	–
BC1608	*Middle AZ hot springs	f__Phormidiaceae_A	6,610,450	46.30	99.37	1.33	N	N	–	–
BC1601	*Upstream main pool	g__Fischerella	5,793,185	40.90	99.04	0.72	Y	Y	+	+
BC1922	Weeping wall cave	g__Fischerella	5,907,625	40.84	97.59	0.72	Y	Y	+	+
BC1902	Red stream	g__Fischerella	5,907,625	40.84	98.31	0.72	Y	Y	+	+
BC1937	Upper source purple	g__Fischerella	5,744,404	40.91	98.55	0.72	Y	Y	+	+

If the strain was grown and tested for far-red light absorption and HPLC UV–Vis to analyze for Chl *f*, the details are listed on the last two columns. If positive, a plus symbol was used to denote positive results, and a negative symbol was used to denote negative results. BC1602 and BC1918 are Group 2 strains that were tested but do not have genome sequences

**Table 2:** Statistics of cyanobacterial environmental metagenome assembled genomes (MAGs) recovered from Arizona Hot Springs

Sample (Temperature ℃)	MAG Name	Lowest GTDB Classification	Size (bp)	GC%	CheckM Completeness %	CheckM Contamination %	FaRLiP?
Purple Stream (43.8)	BC MAG 08	Vampirovibrionales	2,721,736	51.94	92.31	0.85	N
	BC MAG 043	Leptolyngbyaceae	5,460,811	50.34	96.93	0.24	Y
	BC MAG 071	Elainella	4,082,360	51.02	61.85	1.89	Y/IG
	BC MAG 088	Leptolyngbyaceae DSRU01	4,902,443	50.76	95.17	0.59	Y
Red Stream (38.9)	BC MAG 01	Desertifilaceae Roseofilum	4,169,051	46.43	99.45	0.0	N
	BC MAG 023	Cyanobacteriia	4,559,594	53.92	98.11	0.94	N
	BC MAG 038	Leptolyngbyaceae	5,582,962	50.31	99.29	0.47	Y
Green Stream (39.4)	BC MAG 012	Cyanobacteria	4,598,026	53.90	98.35	0.94	N
	BC MAG 027	Phormidiaceae UBA11371	5,868,992	46.33	87.38	2.89	N
	BC MAG 048	Fischerella thermalis	4,183,174	40.94	72	1.69	Y
	BC MAG 089	Leptolyngbyacea DSRU01	4,996,575	50.71	97.52	0.71	Y
Minor Source (44.4)	BC MAG 03	Leptolyngbyaceae DSRU01	5,128,780	50.74	96.23	0.35	Y
	BC MAG 05	Fischerella thermalis	6,181,891	40.97	99.28	0.72	Y
	BC MAG 06	Leptolyngbyaceae	5,860,603	50.28	99.29	0.71	Y
	BC MAG 014	Elainella	4,553,083	40.77	54.03	19.8	N/IG
Purple Source (45.8)	BC MAG 010	Leptolyngbyacea	5,475,957	50.35	98.35	0.24	Y
	BC MAG 026	Leptolyngbyacea DSRU01	5,372,132	50.58	99.53	0.35	Y
Green Source (43.8)	BC MAG 015	Leptolyngbyaceae	5,539,494	50.30	99.41	0.47	Y
	BC MAG 018	Elainellaceae O-77	4,754,583	56.77	92.81	0.94	N
	BC MAG 019	Cyanobacteriia	4,572,174	53.92	98.35	0.94	N
	BC MAG 021	Leptolyngbyaceae DSRU01	5,448,369	50.50	98.58	1.77	Y

Completeness, contamination and GC% were calculated using the program CheckM. The last column determines whether the metagenome assembled has both marker genes that would indicate possible functional potential for FaRLiP acclimation (Y), or not (N). If a genome is less than 70% complete according to the CheckM statistics, incomplete genome (IG) was noted in the FaRLiP column. If both a Y or N was noted with IG, the best possible estimate was given on an incomplete genome

**Fig. 3:**
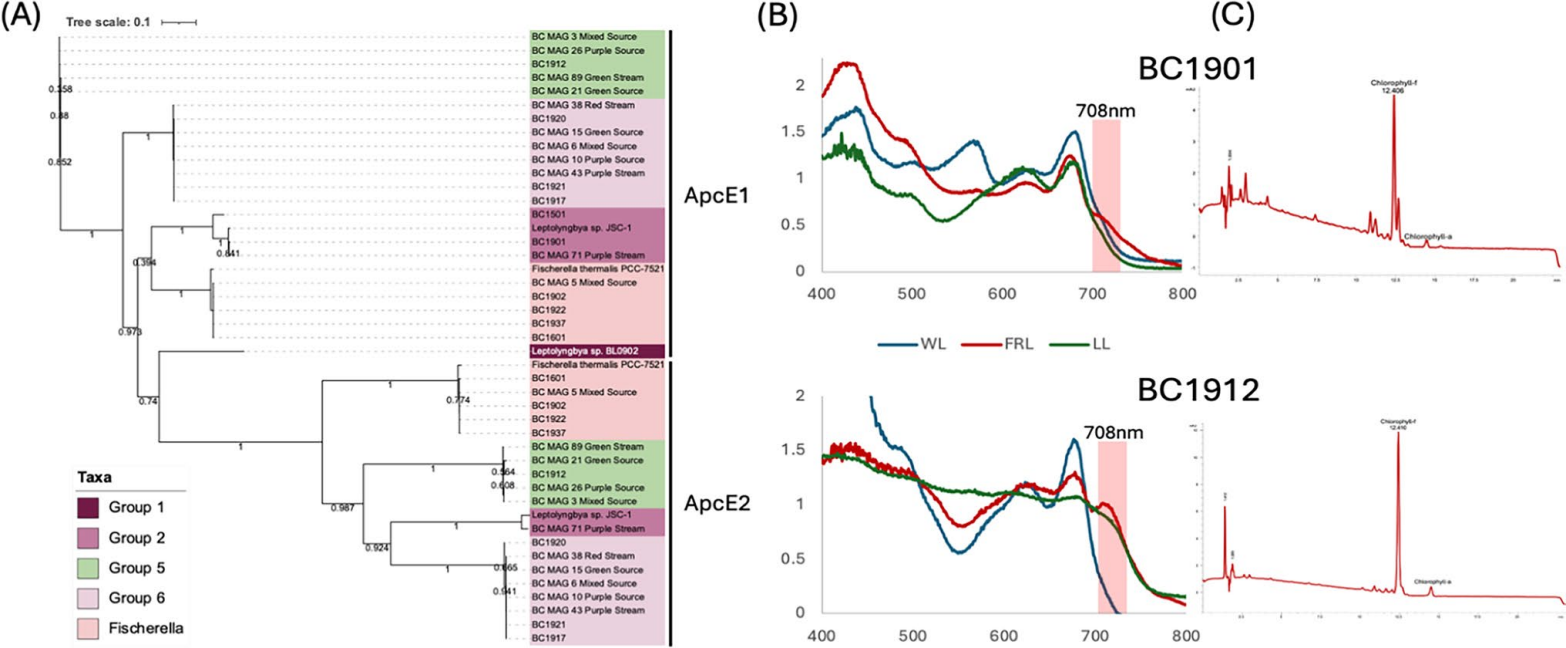
**A** Phylogeny of *ApcE2* relative to *ApcE1*. *ApcE2* is typically used as a single gene marker for Chl *f* production. Black Canyon strain of *Leptolyngbya* BC1901 appears to have only an *ApcE1* gene despite being able to absorb far-red light and produce Chl *f*. **B** The live cell absorbance values detected using a UV–Vis spectrophotometer are shown of the phylogenetic tree. UV/Vis spectra for growth conditions under far-red light as a red line (FRL), low light as a green light (LL) and broad-spectrum light as a blue line (WL) shown for strains BC1901 and BC1912. **C** HPLC pigment analysis of methanol extracts from those cultures indicate presence of Chl *f*

A genome wide phylogenetic tree was constructed by taking 49 gene Clusters of Orthologous Groups (COG) domains as defined by COG (Galperin et al. 2020). These were then used in a multiple sequence alignment for each COG family. Alignments were then trimmed using GBLOCKS and concatenated to construct a phylogenetic tree constructed using FastTree2 version 2.1.10 used with the -fastest setting to estimate maximum likelihood phylogeny. The same tree construction methods were used for assessing the phylogeny of *apcE2*, compared to *apcE1*, its non-FaRLiP homolog of the gene responsible for phycobilisome linker protein production. For gene specific trees and genome wide trees, visualizations of trees were performed using iTOL v6 (https://itol.embl.de/) and can be seen in Fig. 3. Cyanobacteria within the genus *Leptolyngbya* were divided into monophyletic groups with corresponding group numbers. Group number 4 was omitted from this study as it was made up of non-hot spring cyanobacteria. To determine whether the FaRLiP genes were contained in the same operon, OperonMapper software (Taboada et al. 2018) was used on all genome assemblies for cyanobacteria found at Black Canyon. Only results for those containing the cluster of genes are shown in Table 1. *Leptolyngbya* JSC-1 was used as a reference for all other genomes analyzed for operons. Output data from this analysis can be found in Supplemental data S1. OperonMapper annotated gene files were used to create an alignment of the *psbA4/chlF* genes. *Synechococcus* sp. JA-3-3Ab gene *psbA1* (NCBI Reference Sequence: NC_007775.1), a paralog of *psbA4*/*chlF*, was used as the outgroup for this alignment and tree. CLC Genomics Workbench 24 was used to create a Jukes-Cantor Neighbor Joining Tree after 1,000 bootstrap replicates and can be seen in Supplementary Figures S5. OperonMapper provided annotations used in this study are provided in Supplementary data S1. Notable residue differences can be seen in the amino acid alignment used for this tree in Supplemental Figure S8.

The taxonomy of genomes is described by the Genome Taxonomy DataBase v1.6.0 and is listed withinTable 1 (Chaumeil et al. 2019). While BC1917, BC1920, and BC1921 have all been classified as “*Leptothermofonsia*” via this version of GTDB, this group has since been recognized as an invalid name with the correct genus name being *Kovacikia* (Kaštovský et al. 2023; Zampieri et al. 2025).

### Light growth experiments and cell harvesting

Cultures of previously isolated cyanobacteria from the Black Canyon were grown in 50 ml glass culture tubes with 25 mL of BG-11 liquid freshwater media at 30 °C, without shaking. (Moreno et al. 2023). Far-red light growing conditions for the cultures were created by layering two filters, a PNTA GamColor 250 and a PNTA GamColor 660 (https://www.pnta.com), below two FGI far-red LED grow lights (https://forevergreenindoors.com) to filter out all light shorter than 700 nm, with wavelengths centered at 740 nm and a max spectral irradiance of 0.27 W/m^2^ (Bryant et al. 2020). Broad-spectrum lighting and low light conditions were created by layering mesh screen over culture tubes to decrease the strength of the irradiance from broad spectrum LED lights that peak at 634 nm to have a max spectral irradiance of 0.25 W/m^2^, approximately 1.15 µmol photons m^−1^ s^−2^, while the low light conditions had a max spectral irradiance of 0.04 W/m^2^, approximately 0.184 µmol photons m^−1^ s^−2^. Measurements of light quality obtained using a light meter can be seen in supplemental Figure S1.

### In vivo UV–Vis spectrophotometry

The biomass for UV–Vis spectrophotometry and HPLC measurements was obtained by harvesting cells following 30 days of growth, unless noted. Cultures grown under broad spectrum lighting were harvested after 15 days of growth in order to examine cells prior to cell bleaching that occurs by 30 days. To record the absorbance of live cells grown under all light conditions, 5 ml of cell cultures were filtered through 25 mm Whatman glass microfiber filters Grade GF/F and were scanned on an Agilent Cary 300 UV–Vis (Santa Clara, CA) spectrophotometer using a custom set up and typically within an hour of collection, but also on filters frozen at -80 C (Greg Mitchell and Kiefer 1988). A Milli-Q water saturated filter was used as a blank reference prior to performing wavelength scans on each sample. The filters were placed on a custom filter holder that replaces the cuvette holder within the UV–Vis spectrophotometer (Supplemental Figure S7). To gather total ratios of chlorophylls, raw wavelength absorbance values for each strain were first subtracted by the blank measurement and then each resulting value was divided by the average of all absorbance values between 400 and 800 nm (Iturriaga et al. 1988). Strains tested for far-red light absorption include BC1501, BC1502, BC1601, BC1603, BC1608, BC1901, BC1902, BC1903, BC1905, BC1909, BC1911, BC1912, BC1917, BC1920, BC1921, BC1922, BC1930, BC1931, and BC1937, BC1602 (a close relative of BC1501), and BC1918 (a close relative of BC1901).

Three strains were used as controls for UV–Vis spectrophotometry and later pigment analyses. Strain *Leptolyngbya* BL0902 was used as a negative control and was part of our lab’s existing culture collection. *Acaryochloris* RCC-1983, also was part of the our lab’s existing culture collection, was used as a positive control for chlorophyll *d* (Chl *d*) and *Leptolyngbya* JSC-1, purchased from American Type Culture Collection was used as a positive control for Chl *f* after both were grown under far-red light conditions for 30 days (Broun et al. 2010; Mohr et al. 2010).

### HPLC analysis

High performance liquid chromatography was used to identify and distinguish between chlorophylls *d* and *f*. Cells from strains BC1901, BC1912, BC1501, *Leptolyngbya* sp. JSC-1 and *Acaryochloris* RCC-1983 were extracted with methanol to verify the presence of far-red light chlorophylls. 25 mL of culture for each strain were pelleted in a 2 mL microcentrifuge tube at 13,300 xg for 5 min, 1.5 mL of 100% methanol was added and vortexed for 1 min, followed by an incubation period of 30 min with no light at 4 °C. After the incubation period, the samples were centrifuged for 2 min at 13,300 xg and the supernatant was transferred into a glass vial for further analysis. Remaining pellets were discarded and work was performed in regular lab lighting conditions with pigment extracts collected in glass vials wrapped in aluminum foil and stored at -20C until analysis. Extracts were analyzed on an Agilent HPLC system with 1100 G1312A binary pump, 1100 G1315A DAD UV–Vis detector, 1100 G1313A autosampler, and 1100 G1322A degasser (Agilent Technologies, Santa Clara, CA). The system was equipped with a 4.6 × 150 mm Synergy 4µ Max-RP C12 column (Phenomenex, Torrance, CA). The gradient was adapted from Li et al. 2013 and started with H_2_O:MeOH (10:90) that increased to 100% MeOH over 10 min and then was held for 10 min. The solvents were modified with 0.1% formic acid. The chlorophylls *d* and *f* were monitored at 708 nm (Li et al. 2013). Additional strains that had been analyzed using UV–Vis spectrophotometry were also later analyzed using HPLC.

## Results

### Predicting the FaRLiP phenotype using gene homology

Using the characterized gene set from the *Leptolyngbya* JSC-1 FaRLiP gene cluster, Black Canyon metagenomes and cyanobacterial isolate genomes showed the potential for FaRLiP: within the two major cyanobacterial groups (*Leptolyngbya* groups, 5 and 6, isolates listed inTable 1), all genomes appear to have the potential for having the full FaRLiP cluster. Group 2 Black Canyon genomes (isolates BC1901, metagenome BCMAG71 Purple Stream, BC1501) show high homology for some of the FaRLiP genes of the reference strain *Leptolyngbya* JSC-1. BC1501 clearly lacked several important genes such as *psbA3* and *psbA4*/*chlF*, the gene responsible for encoding Chl* f* synthase, and was therefore predicted to not produce Chl *f* (Fig. 2). Within the group of *Fischerella* genomes (Isolates BC1601, BC1922, BC1937, BC1902, and metagenomes BC MAG 48 Green Stream, BC MAG 5 Mixed Source), all appear to have the FaRLiP cluster. This was expected given that the reference genomes, *Fischerella* PCC-7521 and PCC-9605, also all have the FaRLiP cluster as well (Gan and Bryant 2015). All other genomes included in this study lack similar levels of FaRLiP homology relative to those in groups 5 and 6 of the Leptolyngbyaceae groups and the *Fischerella* groups. Strains lacking FaRLiP stand out in Fig. 2, where bright green squares indicate low z-scores.

### Analysis of potential marker genes

While previous studies suggest using ApcE2 as a sole marker gene is sufficient for determining FaRLiP presence, an interesting finding is that BC1901 is missing *ApcE2* and appears to have only an *ApcE1* gene, despite making Chl *f* (see below). This result is important because prior studies have shown that *ApcE2* can be used as a robust marker gene for predicting.

**Fig. 4:**
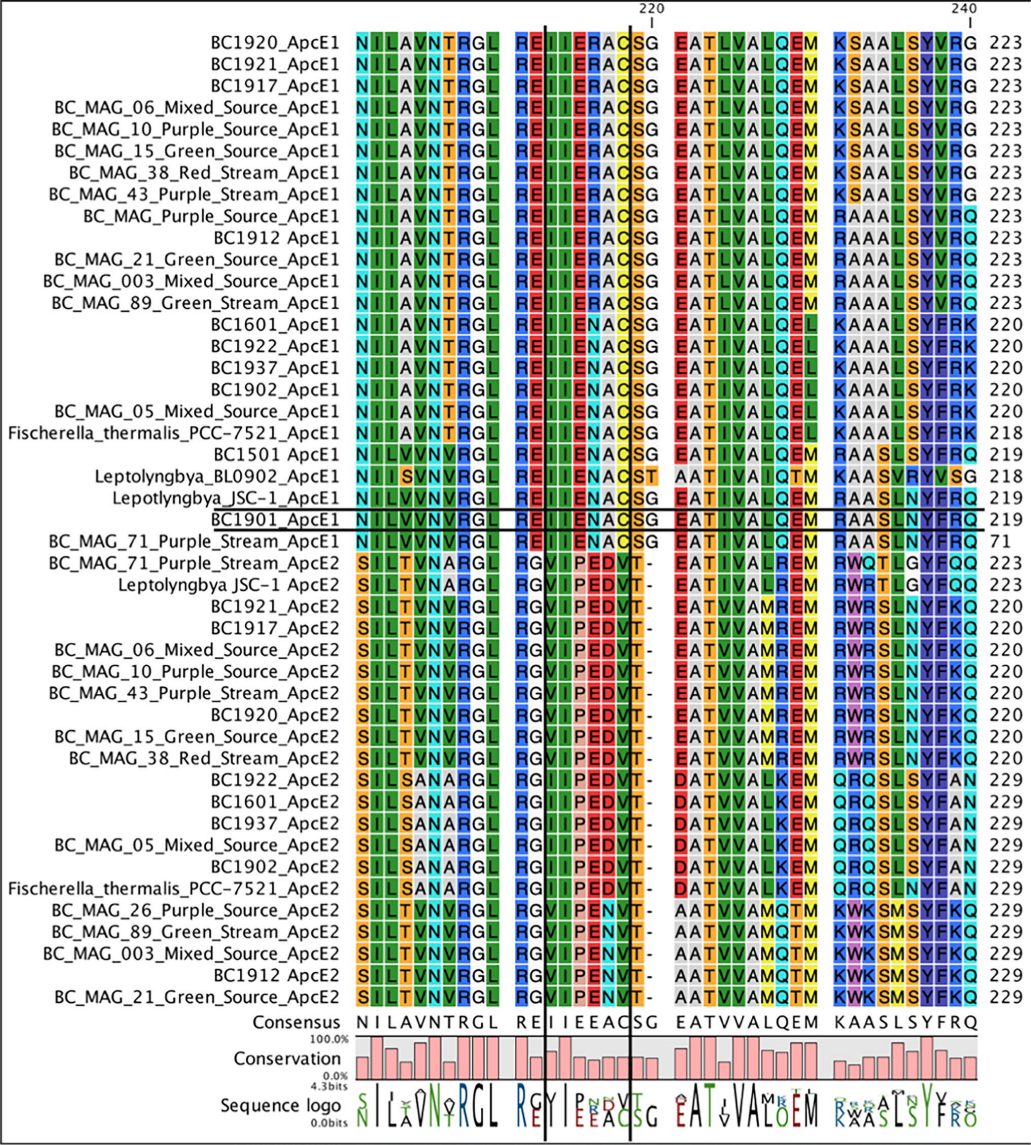
Amino acid residue alignment showing the missing VIPED-like motif in the ApcE1 gene of BC1901 that is typically used as a marker for positive FaRLiP prediction. A modified motif ending in asparagine instead of aspartic acid is seen in BC1912, another FaRLiP cyanobacteria from Black Canyon, at the 217 amino acid position. The results for the rest of the amino acid sequences of *ApcE1* and *ApcE2* in cyanobacterial genomes used in this study can also be seen

FaRLiP which in this case appears to be inconsistent (Antonaru et al. 2020). The missing *ApcE2* gene could be lost in a contiguous sequence during assembly, but the assembly results of the genome show a genome that is 97.8% complete and has less than 1% contamination according to CheckM (Table 1.). We also examined unassembled reads of BC1901 for the *ApcE2* gene but did not find it using both the raw genome sequencing results and by changing the assembly parameters to a minimum contig length of 500 base pairs rather than 2000 base pairs and then reassembling the genomes. When searching raw metagenomic reads for the *ApcE2* gene for BC1901, the results were sequences that look more like an *ApcE1* gene as they were missing the VIPEDV-like motif seen in *ApcE2* (Fig. 4). This approach found reads in the BC1901 genome that were representative of *ApcE1* rather than *ApcE2* and the results were consistent across methods used where contigs were assembled using different parameters, thus leading to the possibility that BC1901 does not have *ApcE2*. Attempts to amplify this gene using previously proposed primers (Antonaru et al. 2020) were unsuccessful in our hands for all *Leptolyngbya* and *Fischerella* strains recovered from Black Canyon and included in this study.

BC1501, another isolated member from that same Leptolyngbyaceae group according to full genome phylogeny seen in Fig. 2, also does not appear to have the *ApcE2* gene, but this was expected given that they also lacked strong homology to the whole gene cluster.

We found the *chlF* gene (a biomarker for FaRLip) in genomes where expected. However we did not detect the expected *chlF* gene in BC1901. It had a paralog compared to that of the model strain, JSC-1. BC1901 was still able to produce Chl *f* as discussed below (Fig. 3). This paralog is also shorter by 30 amino acid residues at the beginning of the amino acid sequence likely due to its location on the end of a contig assembly. After alignment and trimming, the aligned sequences for BC1901 as well as JSC-1 and the rest of the strains in this study, the paralog from BC1901 was shown to cluster together with paralogs from other far-red light absorbers. *Fischerella*-like strains such as BC1902, BC1922, and BC1601 had the full *chlf*F gene discussed previously (supplementary Figure S4). The paralog found in BC1901 was not found in BC1501. It is not unusual to have multiple copies of *psbA-*like genes but it is unclear what is the function of this copy.

The rest of the genes belonging to the FaRLiP cluster can be found in the annotation files for BC1901, where potential FaRLiP genes are found throughout the genome, rather than clustered. There were at most 3 potential FaRLiP genes found per contiguous sequence used to assemble the genome of BC1901, Although the genome reported to have a 97% completion and less than 1% contamination according to CheckM it is still possible that the assembly was poor as we had an unusually high number of contigs compared to the other genomes.

### Pigment detection of live cells using UV–Vis Spectrophotometry

Results for the strains tested are summarized in Table 1 and supplemental Figure S2. Results of light absorption measurements of live cells that in some cases fell below zero may be due to the use of a single filter as a blank before measurements rather than exact matches of those used in each separately measured live cell culture despite all the filters being the same brand and model, ultimately affecting the results even if only slightly. Strains were grown under far-red light for 30 days. BC1912, BC1917, BC1901, BC1601, BC1911, BC1937, BC1921, BC1922 and BC1902, and BC1918 (a close relative of BC1901) all displayed an absorption maximum at about 708 nm when performing a wavelength scan of live cells. Strain BC1920 appeared to have a possible peak at the same range but had lower absorbance values than other strains and to the same strain grown under white light and low light conditions. Negative results for far-red light absorption were seen in the negative control, *Leptolyngbya* BL0902, as well as in BC1931, BC1909, BC1603, BC1911, BC1608, BC1502, BC1903, BC1905, BC1930, and BC1501, and BC1602 (a close relative of BC1501). All strains grown under the low white light conditions did not have UV–Vis absorbance maxima red-shifted further than 700 nm after 30 days of incubation. All strains were also grown under white light as a control and no absorbance peaks were detected at absorbance wavelengths longer than 700 nm in any strains.

### Detection of far-red light absorbing chlorophylls with HPLC

Chlorophyll *f* was detected in the positive control, *Leptolyngbya* JSC-1, with a retention time of 12.41 min. Chlorophyll *d* was also detected in the positive control, *Acaryochloris* RCC-1983, and had a retention time of 12.04 min. HPLC analysis of the Black Canyon strains using the same protocol revealed chlorophyll *f* in BC1901, BC1912, BC1937, BC1917 and BC1921, but not in BC1501—a Group 2 relative of BC1901 and JSC-1 (supplemental Figure S3.). Given that chlorophyll *d* is a minor pigment in FaRLiP strains, a thorough inspection of the HPLC chromatograms revealed trace amounts of Chl *d* in BC1901, BC1912, BC1917, BC1921 and BC1937.

## Discussion

The far-red light photoacclimation gene cluster for FaRLiP has been previously speculated to have originated in a cyanobacterial ancestor after the broad diversification of cyanobacteria and thus is only found in some cyanobacterial lineages (Antonaru et al. 2025). The distribution of these genes is then likely a combination of vertical descent and possibly horizontal transfer, as has been proposed before. In the Black Canyon we found evidence for FaRLiP in the genomes of 9 out of 20 characterized isolates and in 13 out of 21 cyanobacterial MAGs. In our study site, a hot spring in a narrow canyon only about 60 cm wide, FaRLiP was common in the dominant cyanobacteria of the mats, Leptolyngbyaceae groups 5 and 6 as well as those in the group *Fischerella*. These groups accounted for 20 out of the 41 cyanobacterial metagenomes and isolated strain genomes assembled. The evidence of FaRLiP in the site metagenomes shows that the findings are not an artifact of isolate culturing bias.

Group 2 Leptolyngbyaceae showed mixed results for the presence of FaRLiP. Leptolyngbya JSC-1 is a model for the study of FaRLiP (Gan and Bryant 2015). BC1901 lacked strong homology to the marker gene *ApcE2*, but it still appeared likely to have some of the components of the FaRLiP gene cluster scattered throughout the genome as noted by the gene numbers in the Supplemental Data S1 and still produced Chl *f*. BC1901 had FaRLiP matches to the rfpC, psbB2, and 3 copies of the ApcB2 gene, but lacked the rest of the genes necessary to complete the gene cluster. The *rfpA*, *rpfB*, and *rfpC* were found located together in BC1901. We did not find a *chlF* gene in BC1901 highly identical to the one in JSC-1. A paralog was shorter and had residue differences in the 3 conserved locations described previously (Chen et al. 2023). A closely related strain to BC1901 by 16S rRNA (not shown), BC1918 also made Chl *f*.

Another Black Canyon isolate in its group, BC1501 did not produce Chl *f*. A close relative via 16S rRNA, BC1602 isolated a year later also did not produce Chl *f* (not shown). Looking at the genome phylogeny tree in Fig. 2, it is possible that after the divergence of *Leptolyngbya ohadii* and BC1501, the others in Group 2, *Leptolyngbya* JSC-1 and BC1901, gained FaRLiP through a horizontal gene transfer event.

Our difficulty in resolving this HGT event is, as noted above, that *Leptolyngbya* BC1901 appears to be missing (in our genome assembly) an *apcE2* gene and a typical *chlF* gene yet can produce Chl *f* under the laboratory growth conditions used in this experiment. This may be due to the sequencing depth and assembly of this genome. The *PsbA* genes assembled poorly for example and could be why *psbA4* was not annotated when using similar methods that were able to annotate for this gene in the rest of our genomes.

The T-rich motifs from psbA4/chlF seen in previous studies were also seen in all but one, BC1901, of our chl *f* producing isolated cyanobacteria, (Antonaru et al. 2025) (Supplemental Figure S6, data S1). BC1901 and some other strains had a psbA4 paralog of unknown function.

The frequent loss of FaRLiP was noted in the genus *Chroococcidiopsidales* (Antonaru et al. 2023). Detection of only Chl *f* and minute contents of Chl *d*, in all FaRLiP positive strains of this genus was similar to our *various* Black Canyon cyanobacteria which also produce mainly Chl *f* rather than both chlorophylls *d* and *f* at the same concentrations*.* We similarly found that among Group 6, one strain BC1920 had the genes for FarLiP but did not show detectable far-red absorption or Chl *f* (via HPLC), suggesting some strain variability in the Black Canyon as in the *Chroococcidiopsidales*.

Given that filamentous strains of cyanobacteria are thought to be the first to have evolved the ability to produce Chl *f*, it is likely that those characterized in this study can help to understand the evolution of this trait (Antonaru et al. 2025).

The narrow canyon environment is a single factor that could select for FaRLiP, but many strains in the Black Canyon also live in mats. In these systems the cyanobacteria on the bottom of a mat might have an advantage if they could use the light filtered through the mat that might still have near infrared wavelengths. However, group 5 and 6 are the most abundant in the site’s mats (based on metagenomic reads, genomes from these groups make up to as high as 21% of the reads in the Minor Source mat) and both can carry out FaRLiP, so it does not seem that one group has a niche underneath the other group. Mats found in narrow canyon walls such as Arizona Hot Springs, or those found in very shallow cave systems (deep rock overhangs) such as site 3 in Moreno et al 2023, contain a majority of the FaRLiP cyanobacteria. Both environments appear to receive limited direct sunlight. In contrast the genome of BC1502, isolated from an unsheltered hot spring in nearby Boy Scout canyon, did not have the genes for FaRLiP. Future studies that focus on intensity and quality of light throughout the day at these locations would further support the hypothesis that FaRLiP positive strains of cyanobacteria are common in narrow canyon environments and cave-like systems with limited sunlight.

## Supplementary Information

Below is the link to the electronic supplementary material.

Supplementary file1 (DOCX 14814 KB)

## Data Availability

Data is provided within the manuscript or supplementary information files. In addition NCBI BioProject ID PRJNA1419906 contains all genomes and metagenomes discussed in this paper.
