# Supplementary material for: Common capacity for far-red light photosynthesis in a canyon thermophilic freshwater system
Source: Extremophiles. 2026 Feb 21;30(1):11. doi: 10.1007/s00792-026-01422-9 (PMC12924786; doi:10.1007/s00792-026-01422-9)

Appendix

**
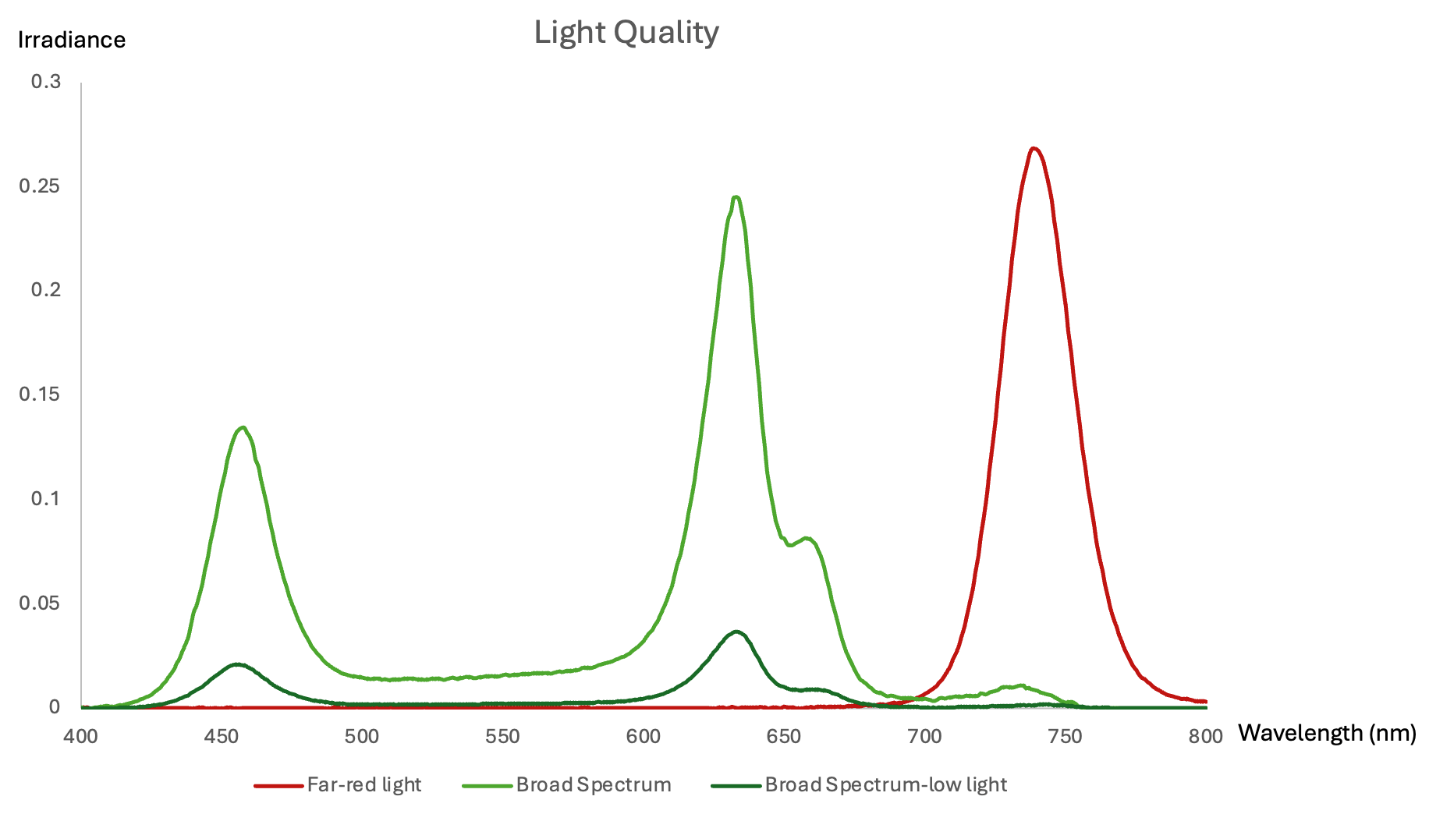
Supplemental Fig. S1** Light intensity and quality across the three growth conditions set up for this study.

**
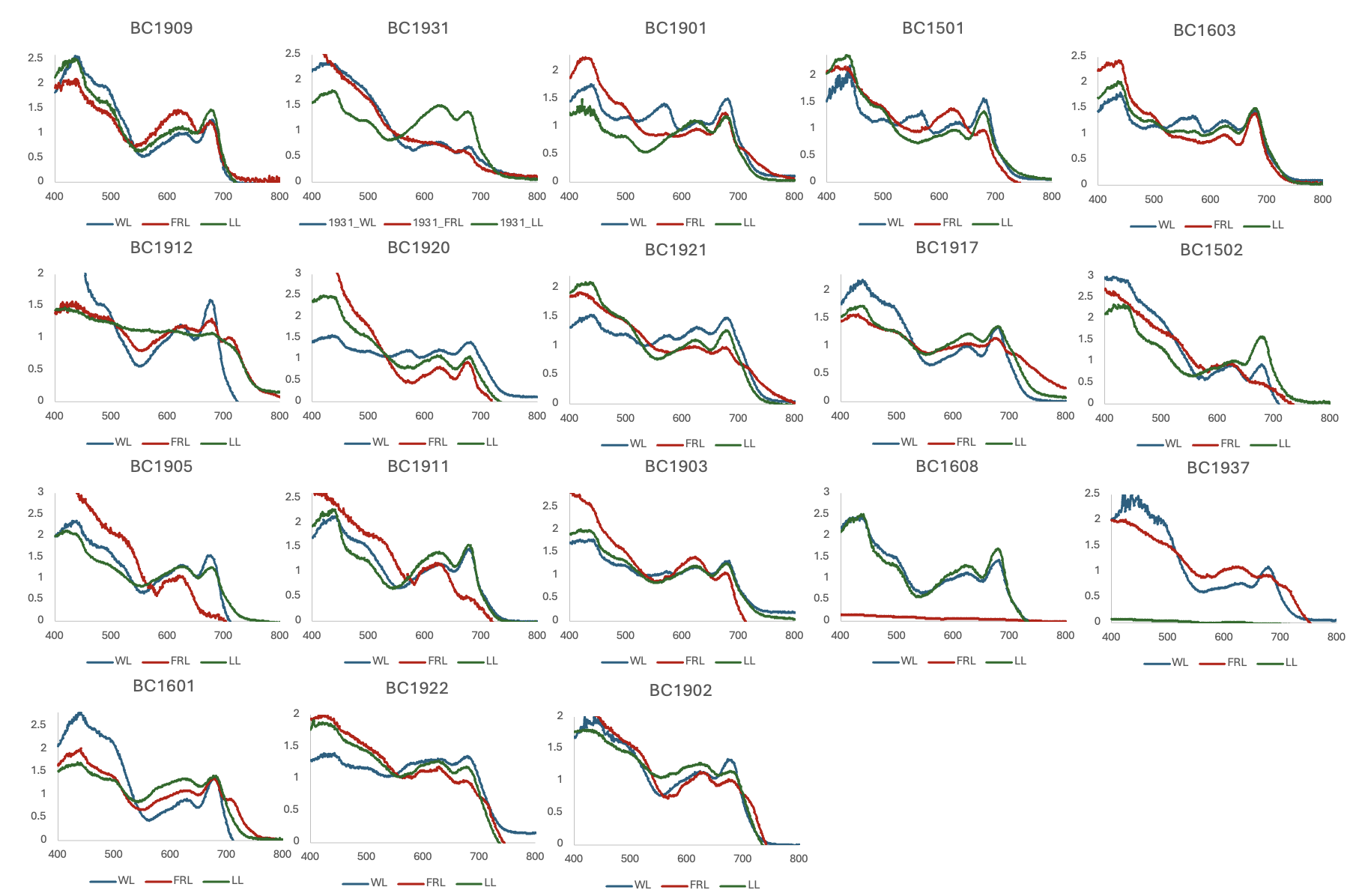
Supplemental Fig. S2** UV-Vis spectra for growth conditions under far-red light (FRL), low light (LL) and broad-spectrum light (WL) for all strains tested for absorbance of far-red light.


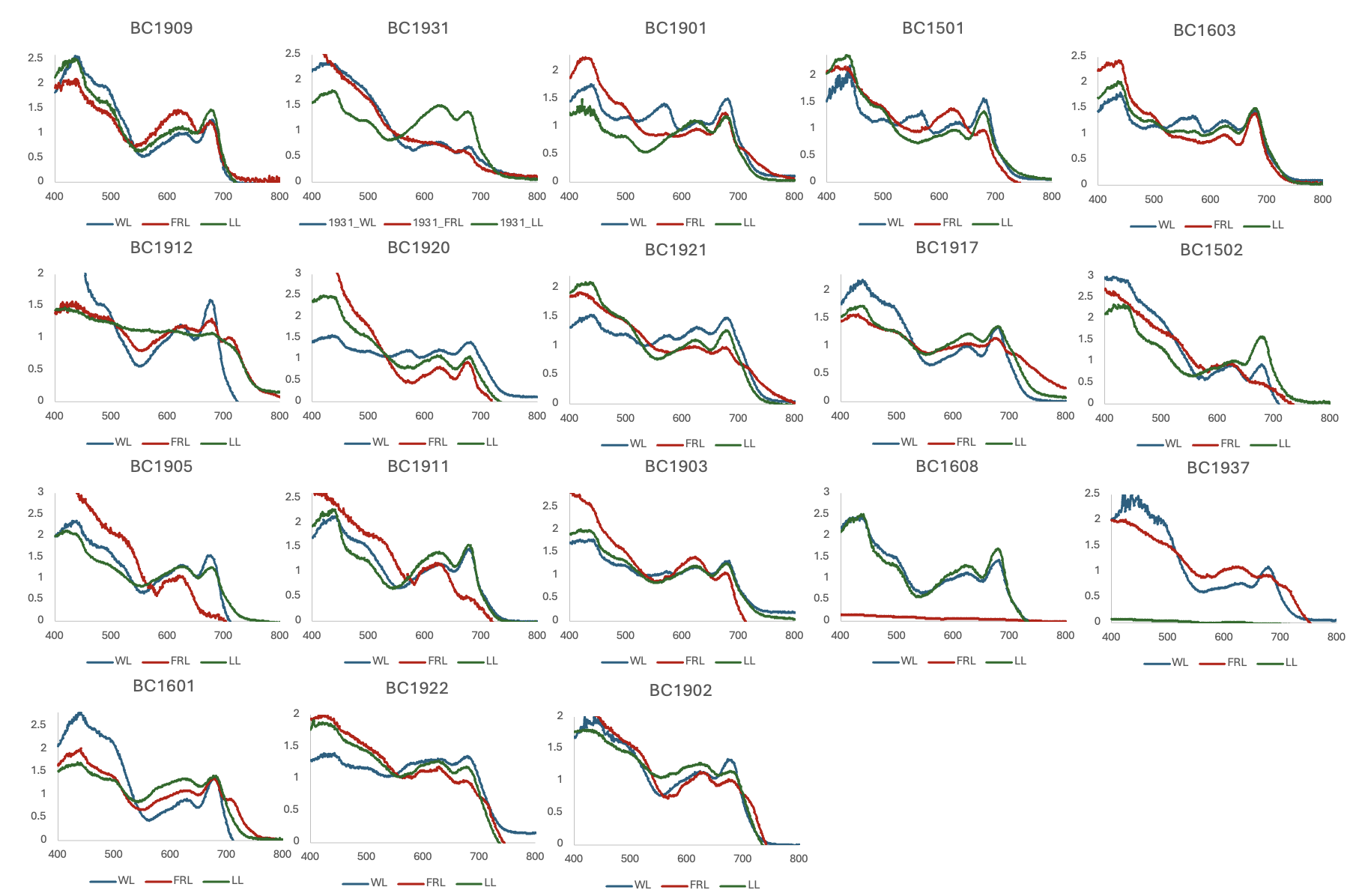

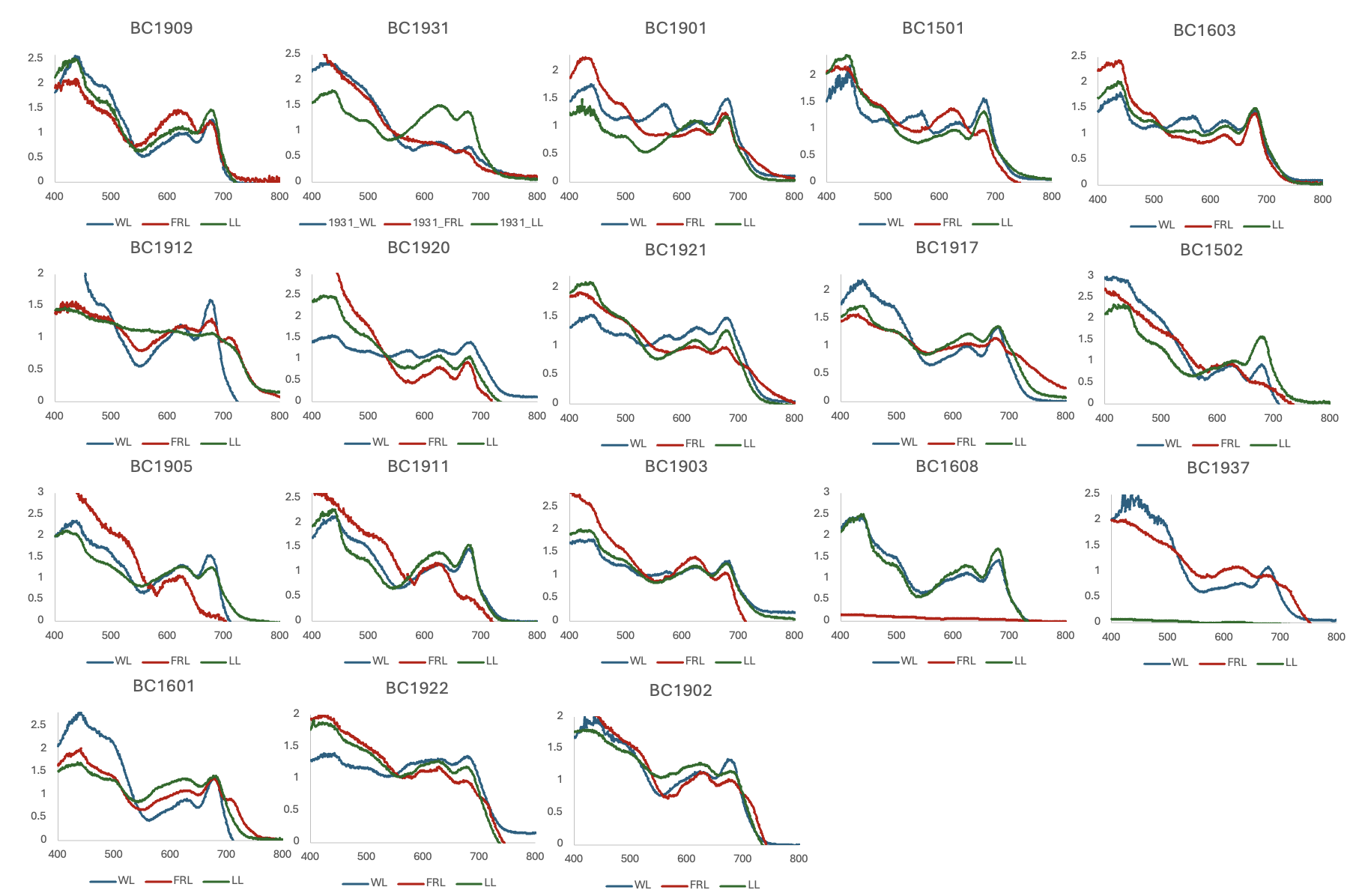

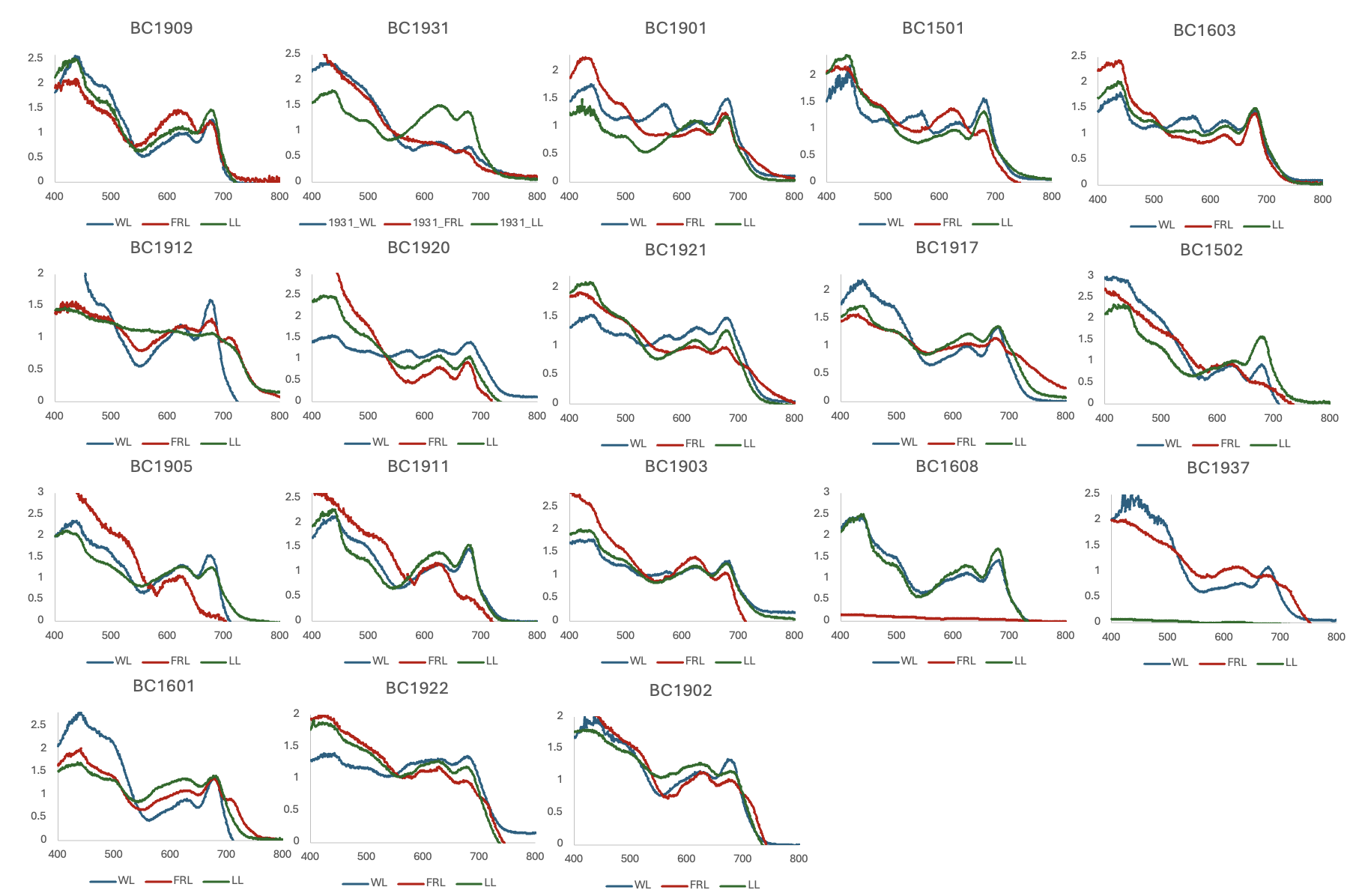

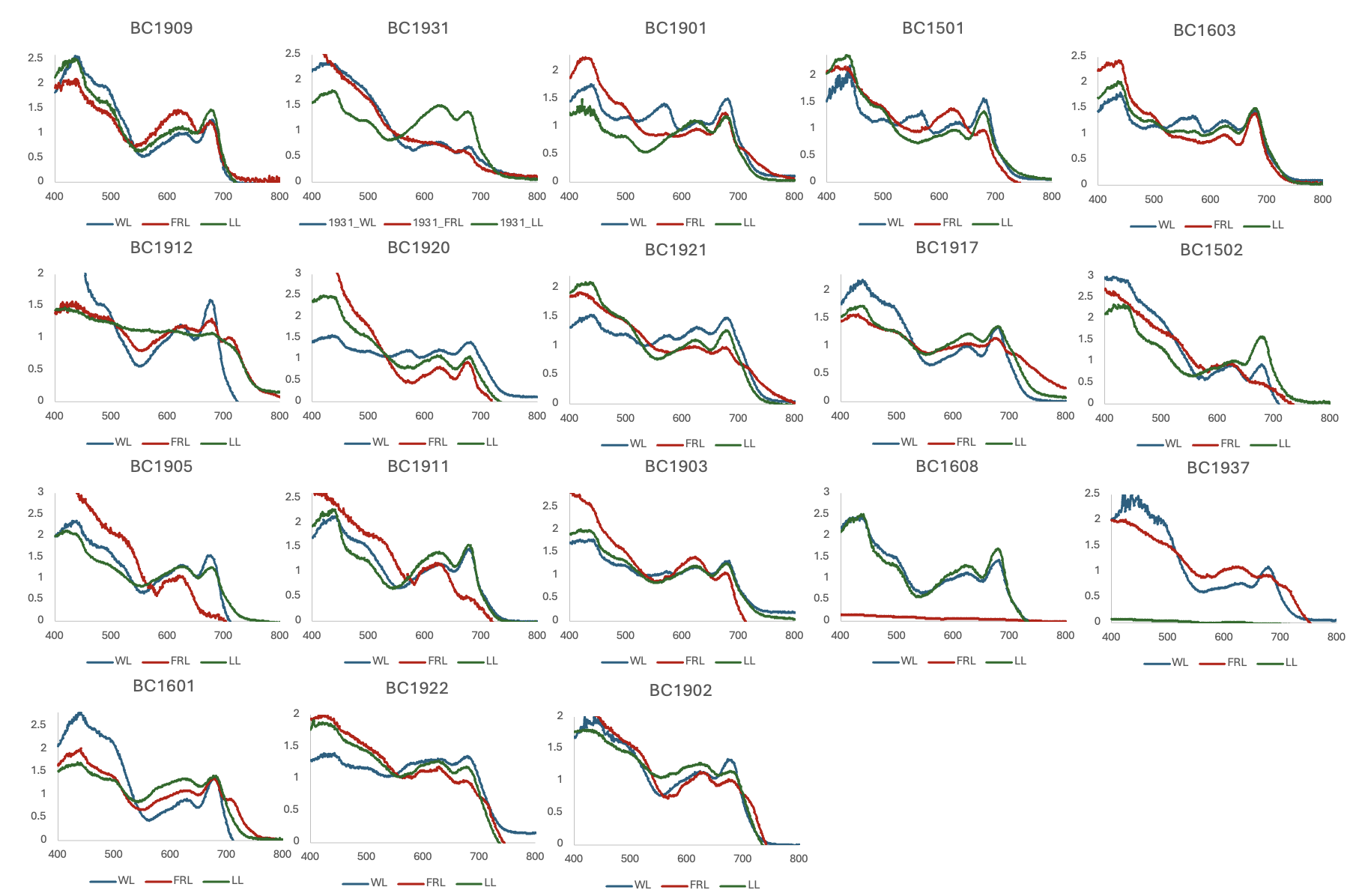

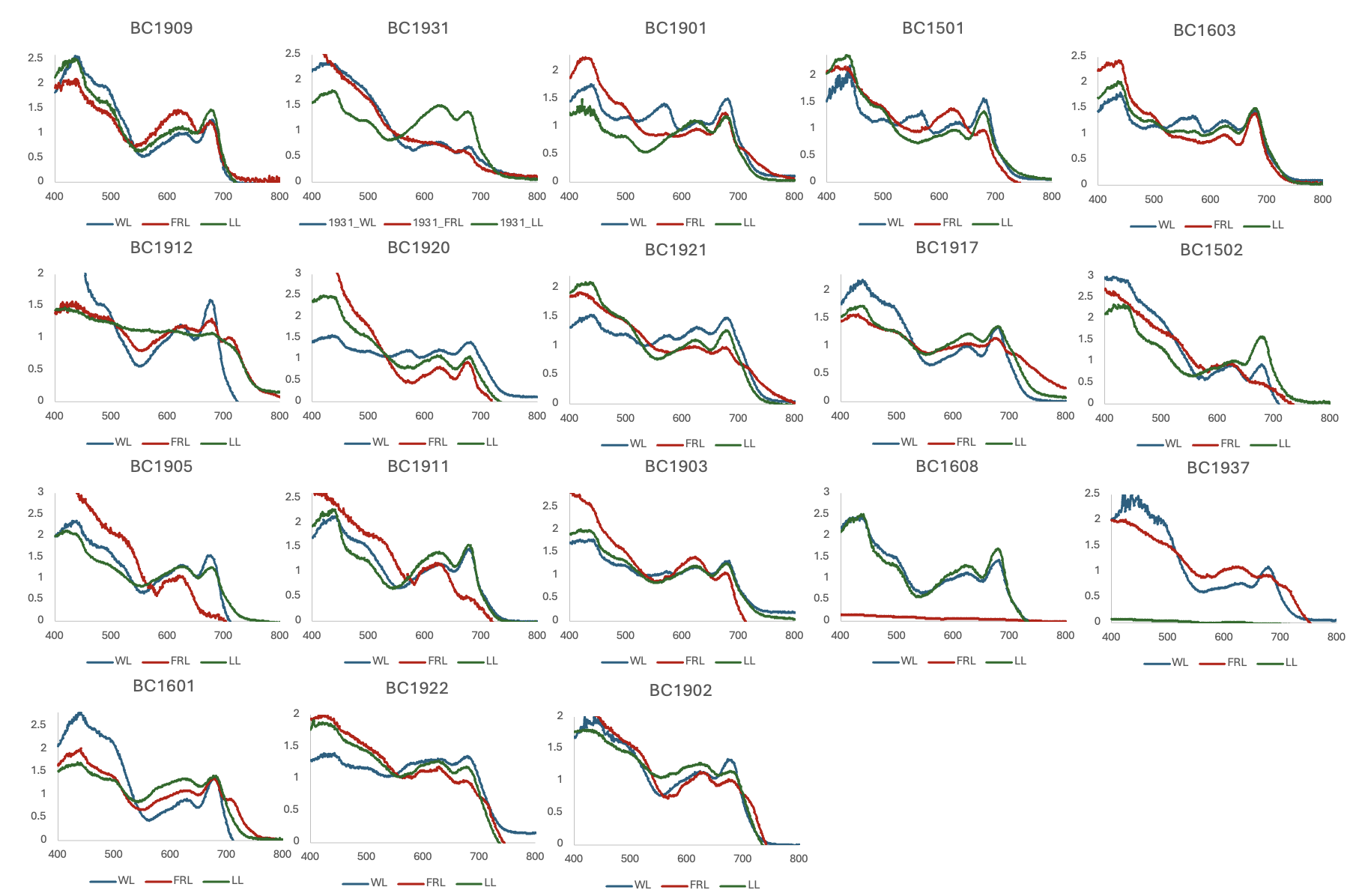


**Supplemental Fig. S3** High performance liquid chromatography analysis of the extracts from the control strain *Leptolyngbya* JSC-1 and Acaryochloris RCC-1983 that produce Chl *f* and Chl *d* respectively. Results for Black Canyon strains BC1501, which didn’t produce Chl *f* and BC1937, which did, also shown in the bottom row. BC1937 had a visible Chl *d* as expected in FaRLiP cyanobacteria.


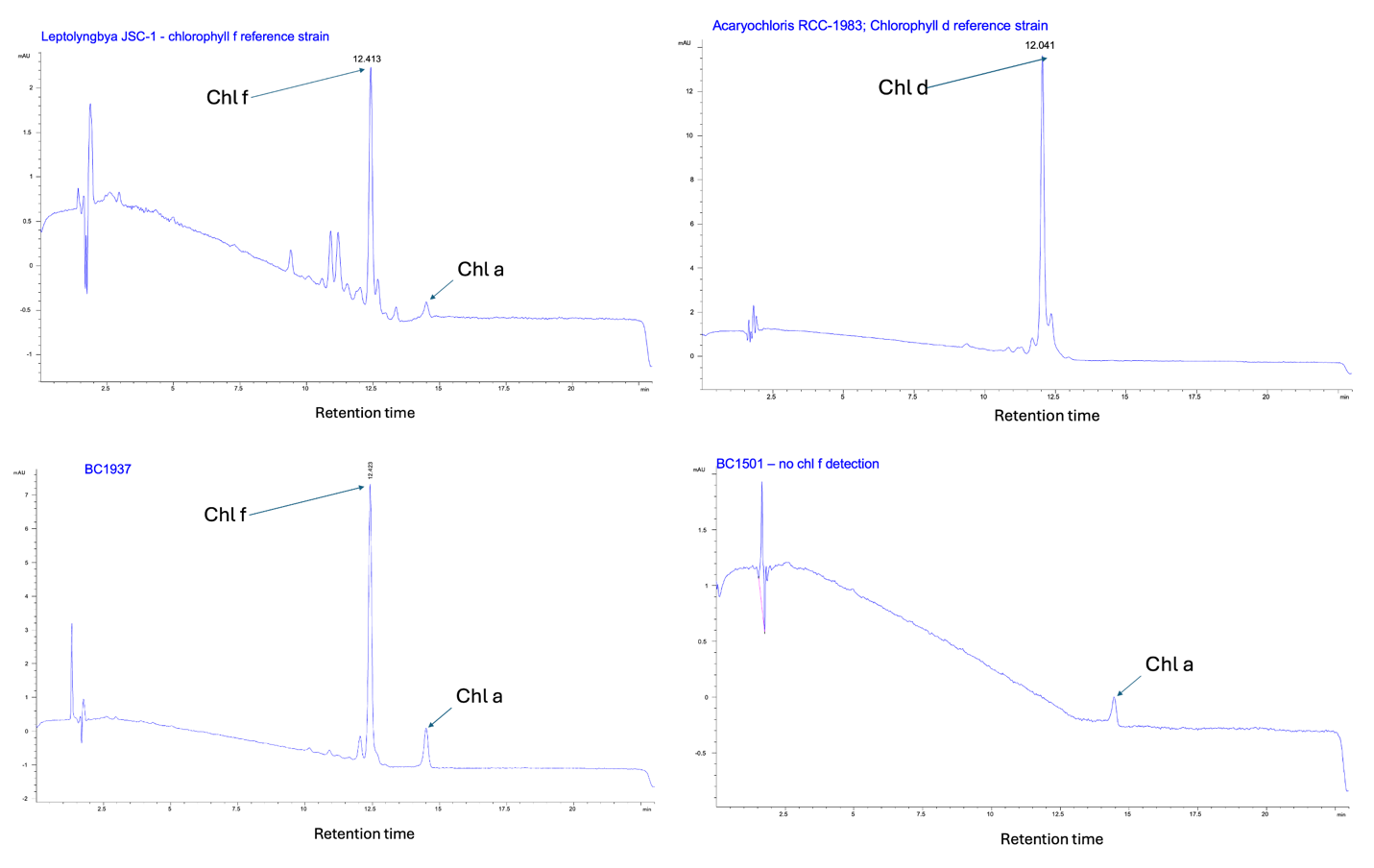

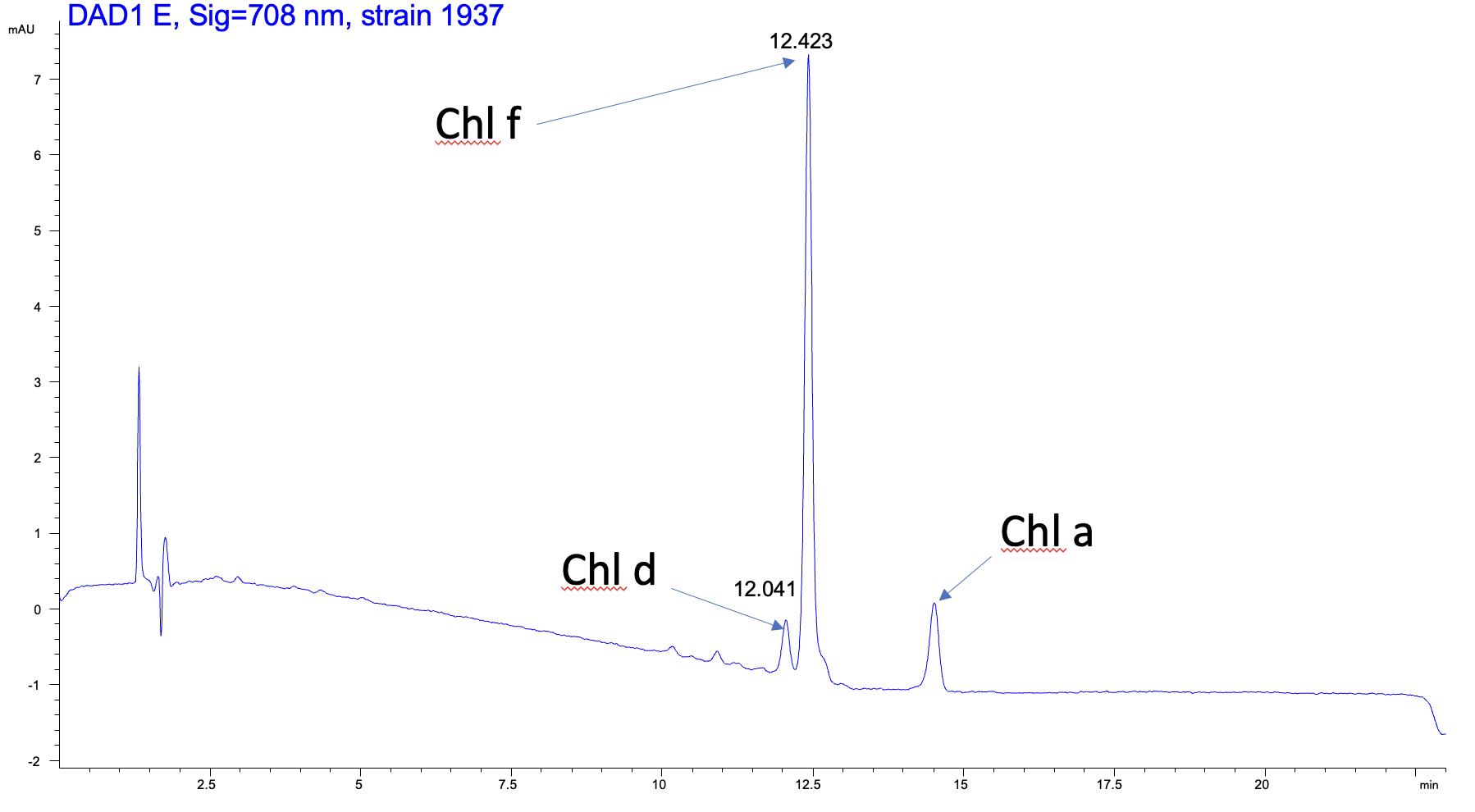


**Supplemental Fig. S4** A phylogenetic tree of matches to Leptolyngbya JSC-1 psbA4 gene created by aligning all BLASTp hits over 60%. The bracket indicates the nodes containing a known psbA4 homolog using the reference to JSC-1. The closest identity to anything resembling psbA4 in BC1901 is also noted with an arrow below.


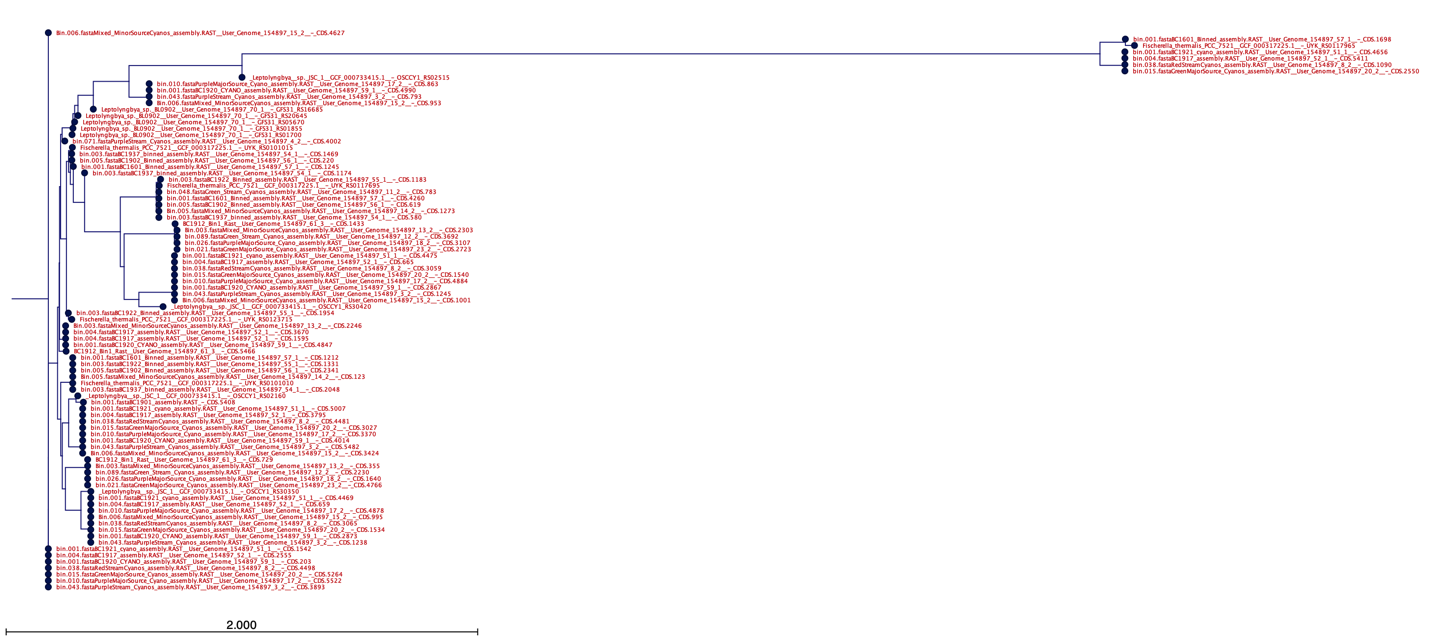


*psbA4*

BC1901

*Chl f*


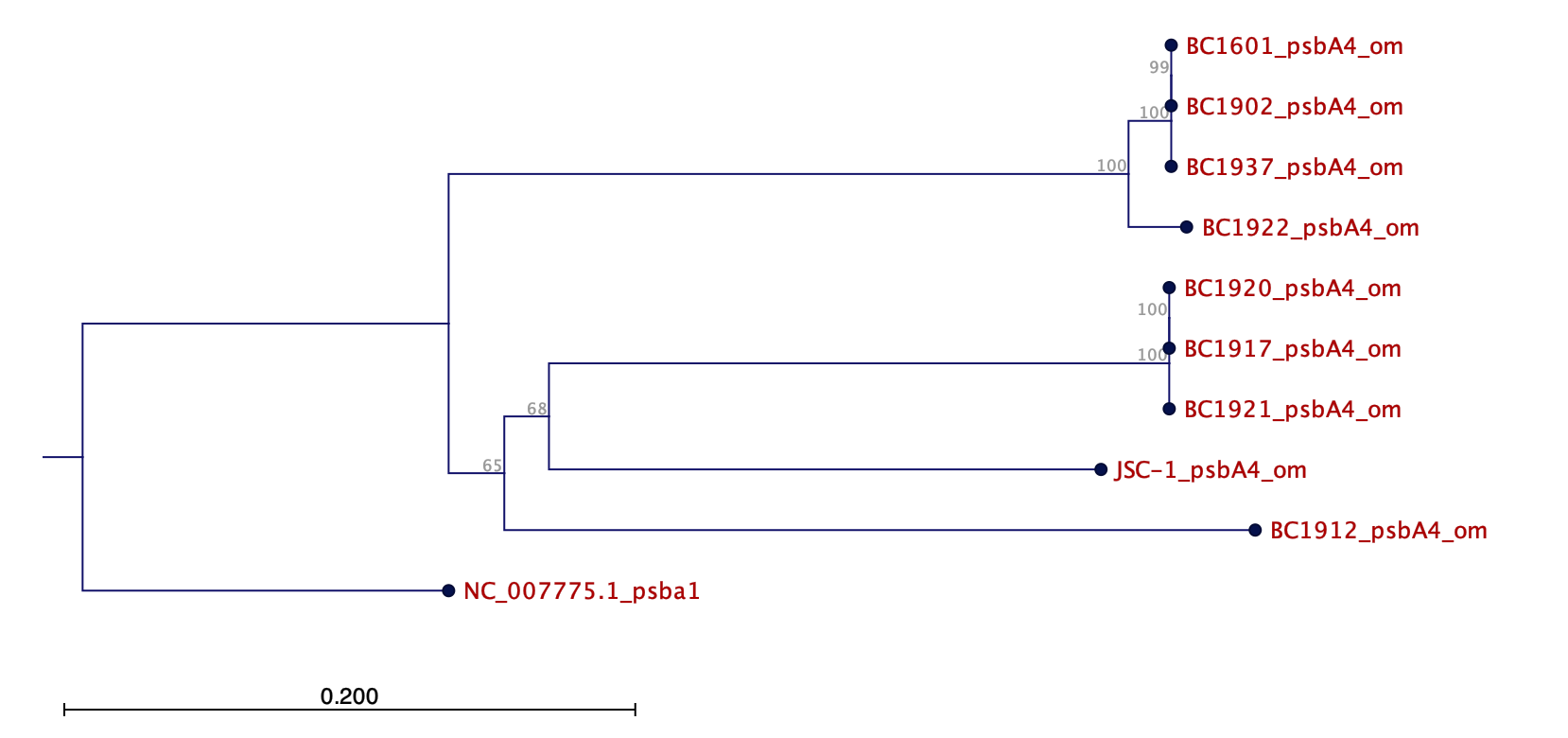
**Supplemental Fig. S5** A phylogenetic tree of matches to *Leptolyngbya* JSC-1 *psbA4* gene created by aligning all retrieved nucleotide genes of *psbA4* using OperonMapper. A paralog of psbA4, psbA1 from Synechococcus sp. JA-3-3Ab, was used as an outgroup for this alignment and tree. The *psbA4* gene is typically found closest to the Group


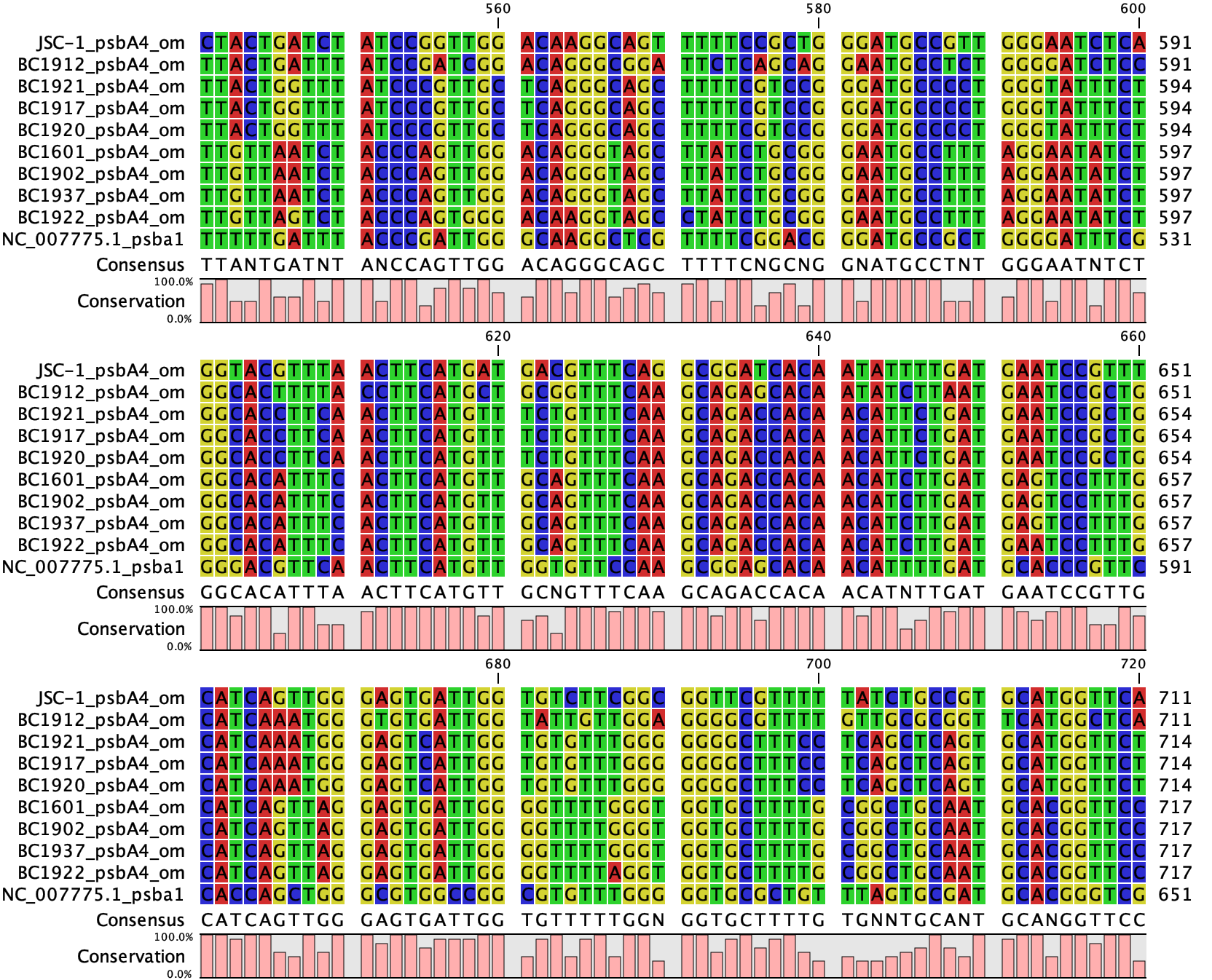
**Supplemental Fig. S6** An alignment describing the T-rich motifs found in FaRLiP cyanobacteria containing the *psbA4/chlf* gene using CLC Genomics Workbench 24.

**Supplemental Fig. S7** (A) Picture of the custom filter holder used to scan the live cells using the UV-Vis spectrophotometer. (B) A picture of spectrophotometer used in conjunction with the custom filter holder.

B

A


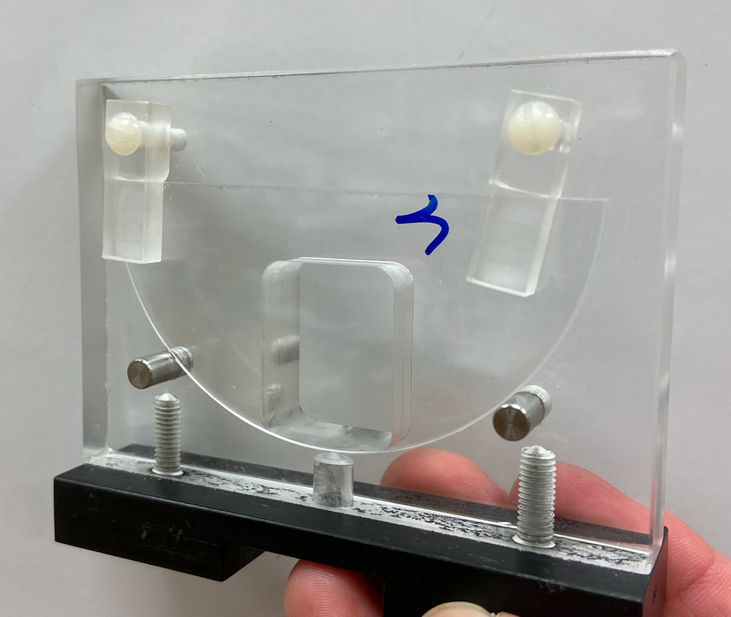

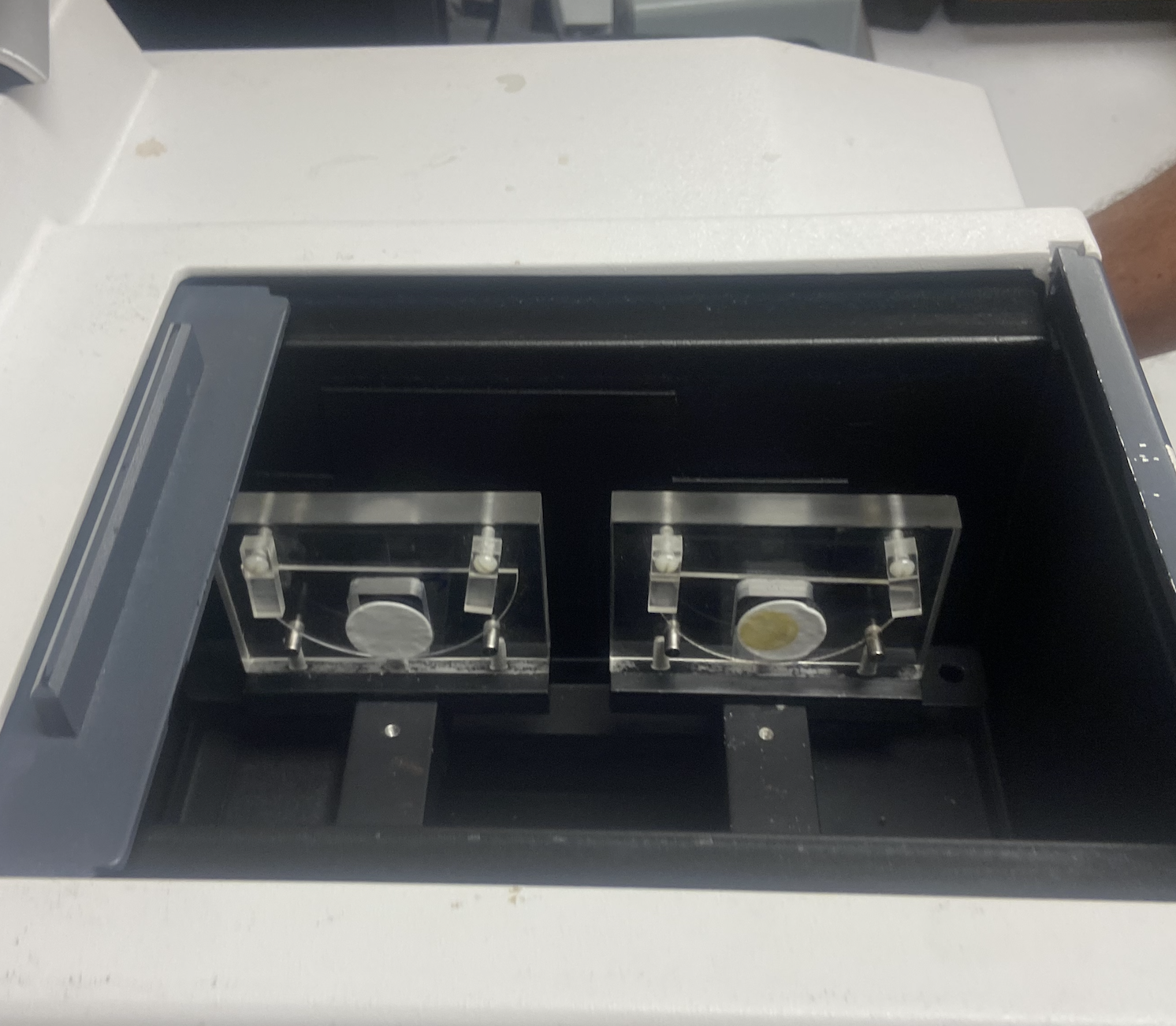


Supplemental Figure S8. An amino acid alignment of the pbaA4/chlf gene as seen in the cyanobacteria included in this study created using CLC Genomics Workbench 24. In red boxes are the three regions previously mentioned as containing residues found in previously described FaRLiP cyanobacteria.


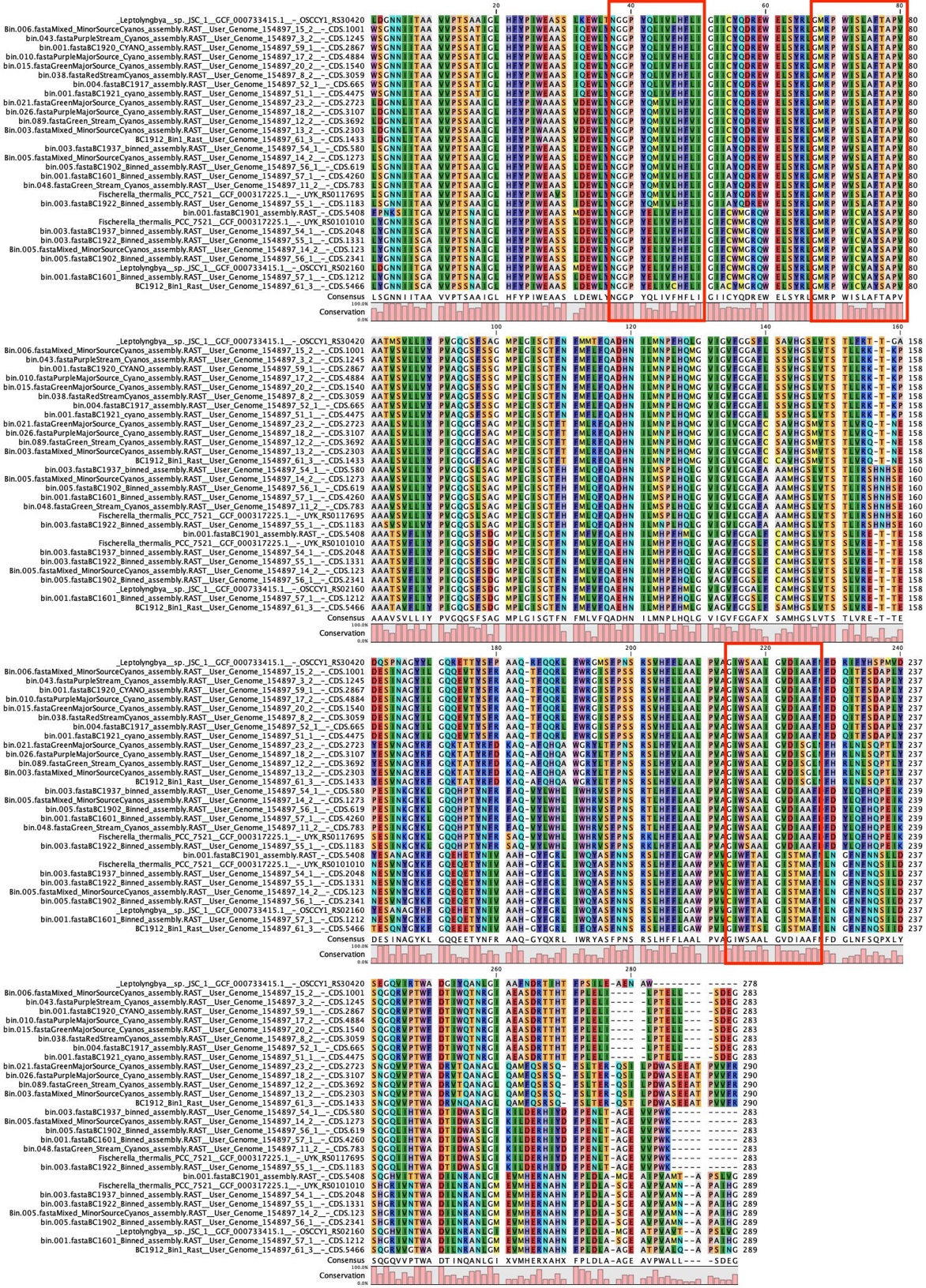

Supplement: Supplementary file 1 — Supplementary file1 (DOCX 14814 KB) [file 792_2026_1422_MOESM1_ESM.docx]
